# Human olfactory mesenchymal stromal cells co-expressing horizontal basal and ensheathing cell proteins in culture

**DOI:** 10.7705/biomedica.4762

**Published:** 2020-03-30

**Authors:** Carlos Ayala-Grosso, Rosalinda Pieruzzini, Leslie Vargas-Saturno, José E. Cardier

**Affiliations:** 1 Unidad de Terapia Celular, Laboratorio de Patología Celular y Molecular, Centro de Medicina Experimental, Instituto Venezolano de Investigaciones Científicas, Caracas, Venezuela Centro de Medicina Experimental Instituto Venezolano de Investigaciones Científicas Caracas Venezuela; 2 Servicio de Neurorinología, Departamento de Otorrinolaringología, Hospital Militar Dr. Carlos Arvelo, Caracas, Venezuela Hospital Militar Dr. Carlos Arvelo Caracas Venezuela

**Keywords:** Olfactory mucosa, homeostasis, mesenchymal stem cells, mucosa olfatoria, homeostasis, células madre mesenquimales

## Abstract

**Introduction:**

The olfactory neuro-epithelium has an intrinsic capability of renewal during lifetime provided by the existence of globose and horizontal olfactory precursor cells. Additionally, mesenchymal stromal olfactory cells also support the homeostasis of the olfactory mucosa cell population. Under *in vitro* culture conditions with Dulbecco modified eagle/F12 medium supplemented with 10% fetal bovine serum, tissue biopsies from upper turbinate have generated an adherent population of cells expressing mainly mesenchymal stromal phenotypic markers. A closer examination of these cells has also found co-expression of olfactory precursors and ensheathing cell phenotypic markers. These results were suggestive of a unique property of olfactory mesenchymal stromal cells as potentially olfactory progenitor cells.

**Objective:**

To study whether the expression of these proteins in mesenchymal stromal cells is modulated upon neuronal differentiation.

**Materials and methods:**

We observed the phenotype of olfactory stromal cells under DMEM/F12 plus 10% fetal bovine serum in comparison to cells from spheres induced by serum-free medium plus growth factors inducers of neural progenitors.

**Results:**

The expression of mesenchymal stromal (CD29+, CD73+, CD90+, CD45-), horizontal basal (ICAM-1/CD54+, p63+, p75NGFr+), and ensheathing progenitor cell (nestin+, GFAP+) proteins was determined in the cultured population by flow cytometry. The determination of Oct 3/4, Sox-2, and Mash-1 transcription factors, as well as the neurotrophins BDNF, NT3, and NT4 by RT-PCR in cells, was indicative of functional heterogeneity of the olfactory mucosa tissue sample.

**Conclusions:**

Mesenchymal and olfactory precursor proteins were downregulated by serum-free medium and promoted differentiation of mesenchymal stromal cells into neurons and astroglial cells.

## Resumen

**Introducción.:**

El recambio celular del neuroepitelio olfatorio ocurre durante la vida del individuo gracias a precursores olfatorios. Además, las células mesenquimales del estroma también contribuyen a la homeostasis de la mucosa. Cuando un explante de una biopsia de mucosa se cultiva en un medio esencial mínimo, se genera una población predominante de células adherentes que expresan proteínas típicas de las células mesenquimales del estroma. La coexpresión de marcadores fenotípicos de precursores olfatorios y de células del recubrimiento del nervio olfatorio constituiría una propiedad única de las células mesenquimales del estroma.

**Objetivo.:**

Determinar si la diferenciación celular de las células mesenquimales hacia fenotipos neurales modula la expresión de los marcadores mesenquimales característicos.

**Materiales y métodos.:**

Se compararon las células aisladas de la mucosa olfatoria en un medio de cultivo con suplemento de 10 % de suero fetal bovino con esferas generadas en un medio sin suero más factores de crecimiento.

**Resultados.:**

Se determinó la expresión de proteínas de las células mesenquimales del estroma (CD29+, CD73+, CD90+, CD45^-^), de las basales horizontales (ICAM-1/CD54+, p63+, p75NGFr+), y de las del recubrimiento del nervio olfatorio (nestin+, GFAP+) en la misma población cultivada. La determinación de Oct 3/4, Sox-2 y Mash-1, así como de las neurotrofinas BDNF, NT3 y NT4, sugirió que las células del estroma son funcionales. La expresión de las proteínas de las células mesenquimales y los precursores olfatorios, disminuyó en las células de las mesenesferas inducidas por ausencia de suero en el medio de cultivo.

**Conclusión.:**

Las células mesenquimales del estroma de la mucosa olfatoria presentan una tendencia dominante hacia la diferenciación neural.

Seminal work from Schwob, *et al.,* has demonstrated that the neuro-epithelium of human olfactory mucosa (HuOM) may be replenished during lifetime by a single multipotent olfactory progenitor cell that occurs in the basal layer of the olfactory epithelium ^1^^,^^2^. Indeed, it was established that globose basal cells (GBC) are the primary progenitors of the OE and play a role as an important source of sustentacular and olfactory sensory neurons (OSN). Additionally, horizontal basal cells (HBC), the second olfactory progenitor, may take the primary role of progenitor once the GBC population is obliterated. Accordingly, the renewal of OE occurs as a result of stringent regulation of cell proliferation and the differentiation by both GBC and HBC olfactory cells ^2^^-^^7^.

Classically, the culture of explants from biopsies of human olfactory mucosa has been performed with an enzyme protease pretreatment which generates a predominant population of mesenchymal stromal cells (MSC), as has been well-established by flow cytometry methodology ^5^^,^^8^^-^^10^. The subsequent expansion of MSCs is performed under *in vitro* culture conditions with fetal bovine serum (FBS) added to the culture medium. As a result of this procedure, olfactory mucosa cells are adherent with fibroblast-like morphology and properties such as proliferation and differentiation which are similar to mesenchymal stromal blood cells from bone marrow ^10^. Although this same embryological origin may provide a similar potential for their application in cellular therapy as those from bone marrow, some differences have been reported ^10^^-^^14^.

The enhanced capabilities of olfactory mucosa MSCs to differentiate to neural tissue probably occur as a result of their ectomesenchymal embryological nature, which has raised great interest for their possible use in regenerative medicine. Therefore, establishing *in vitro* the properties of the olfactory mucosa in tissue biopsies has also proved their efficacy as a source of primary cells for the treatment of neural diseases ^3^^,^^6^^,^^13^^-^^18^.

There is experimental evidence that neural cells obtained *in vitro* from explants of olfactory mucosa may be used for regenerative purposes ^11^^,^^12^^,^^14^^,^^19^^-^^22^. Recent evidence has shown that human olfactory mucosa stromal cells (SC) may offer unique properties as a peripheral reporter in some neuropsychiatric disorders ^23^^-^^27^ and chronical diseases such as Alzheimer's ^28^^,^^29^ and Parkinson ^30^.

Taking into consideration the potential of MSCs for cell transplantation, several authors have pointed out some issues regarding the use of FBS for therapeutic applications and research. For instance, variability between experimental results has been reported due to the complex formulation of serum and the inconsistency between the lots ^15^^,^^31^^,^^32^. In this sense, it is important to develop better-defined media without serum which may modulate the metabolic machinery of cells and, in some cases, the expression of characteristic proteins ^9^.

Given that the olfactory mucosa is formed by multiple types of cells, it is likely that *ex vivo* preparation under culture conditions may be a source of olfactory progenitors, ensheathing cells, and olfactory sensory neurons. Accordingly, establishing the appropriate culture conditions for the proliferation of mesenchymal stromal, olfactory progenitors, and ensheathing cells from tissue explants, and their differentiation in neural cells may offer comprehensive knowledge for cell transplantation.

In the present study, we asked ourselves whether the expression of olfactory mucosa MSC proteins could be modulated by serum-free conditions in the culture medium. To check it, we determined the expression of proteins of mesenchymal, olfactory progenitors, and ensheathing cells in mesenchymal neurospheres that are the predominant proliferative form under serum-free conditions. Neuronal and glial differentiation was preferred with a serum-free medium suggesting a neuron-glial-oriented differentiation program of olfactory stromal cells.

## Materials and methods

### Isolation and culture of cells from human olfactory mucosa

All the procedures of the study were approved by the Institutional Ethical Committee and it was conducted in compliance with the Declaration of Helsinki for Medical Research Involving Human Subjects. Human olfactory mucosa samples were isolated from upper turbinate nasal mucosa biopsies collected from patients by endoscopy as previously described ^33^. Briefly: The procedure was performed by an otorhinolaryngologist under local nasal anesthesia (lidocaine liquid 4%, oxymetazoline HCl 0.05%). For the endoscopic procedure, we used a biting forceps for tissue sampling. Following the collection of the tissue biopsy, subjects remained under observation for 15 min to ameliorate any discomfort. Biopsies of 3 to 6 mm^3^ tissue samples were transported in DMEM/F12 medium with 2% penicillin/ streptomycin and kept between 4 and 8 °C until dissection. Tissue samples were further dissected in two fragments: One was fixed in 4% p-formaldehyde for histological studies using regular H&E histochemistry and immunostaining while the second fragment was used for generating tissue explants of 0.5 to 1 mm^2^ using micro scissors under sterile conditions. One to three explants were placed in each of the 24 wells of the plate with DMEM/F12 culture medium supplemented with 10% FBS, 0.5% penicillin/streptomycin, and glutamine (DMEM/F12-CM) in a CO_2_ incubator.

### Cell culture reagents

The Dulbecco Modified Eagle Medium (DMEM/F12) culture medium was obtained from GIBCO (NY, USA), the fetal bovine serum (FBS) from SIGMA (St. Louis, MO, USA), the TrypLE Express and the N2 supplement from GIBCO, the recombinant human basic fibroblast growth factor (b-FGF, R&D systems, 234FSE), the recombinant human epidermal growth factor (EGF, GIBCO, PHG0311), and Forskolin (F6886) and Trizol reagents were obtained from SIGMA (St. Louis, MO, USA). The antibodies for immunofluorescence fluorescein isothiocyanate (FITC)- or phycoerythrin (PE)-conjugated monoclonal anti-human antibodies CD29, CD54, CD73, CD90, nestin, and CD45 were purchased from Becton Dickinson (San Diego, CA, USA); CD271-FITC was from Biolegend (Minneapolis, MN, USA), GFAP from DAKO, and class III p tubulin, from Millipore (table 1).

**Table 1:** Antibodies and cytokines

Antibody	Species	Dilution	Company	Catalogue #	Antigen
					
FITC Isotype	Mouse	1:200	Biolegend	400110	--
PE Isotype	Mouse	1:330	Biolegend	400114	--
CD29 PE	Mouse	1:160	BD Pharmigen	555443	Human
CD54 PE	Mouse	1:160	BD Pharmigen	555511	Human
CD73 PE	Mouse	1:160	BD Pharmigen	555257	Human
CD90 PE	Mouse	1:160	BD Pharmigen	555596	Human
Nestin PE	Mouse	1:160	BD Pharmigen	IC1259P	Human
CD271 FITC	Mouse	1:160	BD Pharmigen	345104	Human
GFAP	Rabbit	1:160	DAKO Cytomation	Z-0334	Human
TUJ-1	Mouse	1:160	SIGMA	T-8660	Human
P63	Rabbit	1:160	BSB-5851	BSB-5851	Human
CitK5/6	Mouse	1:160	Millipore	MAB1620	Human
HuR EGF		20 ng/ml	GIBCO	PHG0311	Human
HuR FGF		20 ng/ml	GIBCO	234 FSE	Human
Forskolin		5 µM	SIGMA	F6886	
Α Transretinoic acid		1 µM	SIGMA	R2625	

### Osteogenic, chondrogenic, and adipogenic differentiation of human olfactory mucosa MSCs

The multipotential capacity of olfactory mucosa MSCs was examined by culturing cells in osteogenic, chondrogenic, and adipogenic differentiation media as previously described ^17^^,^^34^. Briefly, the olfactory mucosa MSCs from passages 2 to 5 cultured in DMEM/F12-CM medium were harvested using TrypLE express solution and seeded at 250,000 cells/cm^2^ in a 24-well plate in osteogenic, chondrogenic, and adipogenic differentiation medium. Cells were kept under these conditions for 21 to 28 days replenishing the medium every 4 to 5 days. Once the differentiation endpoint was reached, the cell culture was washed with PBS and fixed in 10% p-formaldehyde. Alizarin red, alcian blue, and oil red histochemical stainings were used to reveal calcium deposits for osteogenesis, glucosamine and glucan for chondrogenesis, and cytoplasmic fat drops as adipogenesis indicators, respectively. Bright-field photomicrographs were obtained with a Zeiss Axiovert Observer D1 inverted microscope equipped with a Tucsen ICC 5.0 ICE digital camera routed to a computer.

### Flow cytometry analysis of olfactory mucosa stromal cells

Adherent cells generated from explants began to proliferate and migrate from the tissue. A crown of cells surrounding explants became confluent after 7 to 10 days in culture. Cells were collected and transferred to plates with a larger growing area until passage 2 to 5 for analysis. Phenotypic characterization of cells was done using the flow cytometry methodology for the expression of extracellular MSC (CD29, CD45, CD73, CD90) proteins and intracellular ensheathing and neural progenitors (nestin, glial fibrillary acidic protein (GFAP, DAKO), HBC (ICAM-1/CD54, p63 (DAKO), and low-affinity neural growth factor (FITC-NGFr/p75) (BioLegend®) proteins. Simultaneous negative control staining reactions were performed by incubating the cell preparation with the corresponding IgG isotype FITC or PE derivative. In case no conjugated primary antibody was used, the cell preparation was analyzed using the secondary antibody as the negative control.

Intracellular labeling was performed with the Cytofix™/Cytoperm™ BD (554722) kit and the Becton and Dickinson datasheet instructions. Briefly: 350 ul of Cytofix™ were applied to 10^6^ cells and kept on ice for 20 min. Then 450 |l of Cytoperm™ (1/10 dilution with water) and cell suspension were centrifuged and re-suspended in a volume of Cytoperm™ sufficient for all determinations. The data collection and the analysis of the fluorescent intensities were made using the FACS Calibur™ platform (Becton Dickinson, San Jose, CA). Ten thousand events were acquired and analyzed using the BD Cell Quest™ software program.

### Detection of transcription factors and neurotrophin indicatives of olfactory progenitors and ensheathing/neural progenitor stromal cells by RT-PCR

Total RNA was extracted from 90% confluent olfactory mucosa MSCs in DMEM/F12-CM using Trizol according to the manufacturer's instructions. Reverse transcription was carried out using random hexamer oligonucleotides and 4 U AMV reverse transcriptase (Promega, Madison, WI) for cDNA synthesis. The RNA quality was assessed using the Agilent 2100 Bioanalyzer (Foster City, CA) and quantified with a NanoDrop spectrophotometer (Wilmington, DE). PCR amplification of the cDNA was then performed using Platinum™ Taq DNA polymerase (Invitrogen™) and specific oligonucleotides (table 2) for the detection of transcriptional factors Oct 3/4, Sox-2, and *Mash-1* and neurotrophins; brain-derived nerve factor (BDNF); neurotrophin 3 (NT3) and neurotrophin 4 (NT4), and B-actin transcripts from 40ng of RNA equivalent cDNA with a blank RT control. PCR conditions were subjected to a denaturing step for 2 min at 94 °C, followed by 35 cycles of 1 min at 94 °C, annealing for 1 min at 50 °C (Mash-1), 52 °C (Oct 3/4, NT3, and NT4), and 54 °C (Sox-2 and BDNF), and an extension for 1 min at 72 °C. The analysis of the PCR products was performed by comparing them with the predicted PCR fragment size after their staining in ethidium bromide and separation by electrophoresis in a 1.8% agarose gel.

**Table 2:** Primer sequences specific for neural growth factors

Primer	Sequences	Base pairs
		
Oct –	5'-GAGCAAAACCCGGAGGAGT-3'	310
	5'-TTCTCTTTCGGGCCTGCAC-3'	
MASH-1 5'-GCGTTCAGCACTGACTTTTG-3'		207
	5'-CCCCGGGAGACTTCTTAGAG-3'	
Sox-2	5'- CGGCCCCGGCGGAAAACCAA-3'	515
	5'- TCGGCGCCGGGGAGATACAT-3'	
BDNF	5'- AGCCTCCTCTGCTCTTTCTGCTGGA-3'	298
	5'- CTTTTGTCTATGCCCCTGCAGCCTT-3'	
NT4	5'- AGCGAAACTGCACCAGCGAG-3'	202
	5'- CACCTTCCTCAGCGTTATCA-3'	
NT3	5'- CCCGAGAGCCGGAGCGGGGA-3'	230
	5'- GTGACTCTTATGCTCCGCGT-3'	
β-actin	5'- TCCTGTGGCATCCACGAAACT-3'	340
	5'- GAAGCATTTGCGGTGGACGAT-3'	
		

### Adaptive response of olfactory stromal cells to culture medium conditions

All experiments under serum-free conditions were performed with cells from passage 3 to 5 grown in DMEM/F12-CM culture. Once cells became confluent, the culture medium was replaced with a DMEM/F12 serum-free medium. Then, after five days, cells were collected by TrypLE Express solution treatment and transferred into a serum-free DMEM-F12 culture medium containing N2 supplement with Hu-EGF (20 ng/ml) and b-FGF (20 ng/ml) (DMEM/F12-GF) added to the medium.

Under DMEM/F12-GF culture conditions, mesenchymal neurospheres occurred after 2 days and proliferated or not as spheres of low-adherent stromal cells. After 7 days, the mesenchymal neurospheres were harvested, dissociated after incubation with TrypLE Express solution, and characterized by flow cytometry. Their functional properties were studied under the specific conditions described below.

### Cells from mesenchymal neurospheres induced by serum-free DMEM-GF

*Flow cytometry analysis:* Mesenchymal neurospheres induced by DMEM-GF culture conditions were collected and dissociated to single cells after incubation with TrypLE Express solution for 15 min. Cells were labeled directly with the conjugated primary antibody (3 µl for 10^5^ cells, plasma membrane immune labeling) or fixed and permeabilized with a Cytofix™/ Cytoperm™ permeabilization kit. Then, cells were incubated for 30 min with conjugated primary antibodies or the corresponding isotype. After incubation with the antibody, each tube was rinsed with PBS and centrifuged at 1500 rpm for 5 min. In each experiment, the percentage of immunopositive cells was determined using the corresponding isotype or the secondary antibody with no primary negative control.

### Neurogenic differentiation of cells from mesenchymal neurospheres induced by serum-free DMEM-GF culture conditions

Spheres obtained under DMEM/F12-GF conditions were examined for their neurogenic potential by culturing single cells in neural differentiation media. Briefly, single cells were seeded at 3.5 x10^4^ cells/cm^2^ in a 24-well plate on coated coverslips with poly-L-lysine (10 µg/ml) solution. Our protocol for elevating intracellular cAMP was modified from Deng, *et al.*^8^. In addition to the primary culture conditions to generate mesenchymal neurospheres, single cells were differentiated in serum-free DMEM/F12 plus N2, α-trans retinoic acid (1 µM), R-2625, SIGMA), and forskolin (5 µM, F6886, SIGMA) by 7 days after plating (DMEM/F12-RA+FORSK). Forskolin, a phosphodiesterase inhibitor, increases cAMP levels and retinoic acid induces neural differentiation. The DMEM-GF medium was changed on day 4 with a freshly prepared medium. On day 7, the cell culture medium was removed and cells fixed with p-formaldehyde 4% in PB 0,1 M for 15 min. Once fixed, plates were maintained in PBS solution at 4 °C until immunostaining was performed.

### Immunostaining of dissociated mesenchymal neurosphere growth on coverslips

Cells on coverslips were permeabilized with PBS (0.01 M) containing 0.1% Triton X-100 at RT for 30 minutes. Nonspecific sites were blocked with 2% normal goat serum (Invitrogen™) for 30 minutes. Cells were then incubated with the primary antibody TUJ-1 (1/200 Mouse monoclonal tubulin p-III antibody, Millipore) and GFAP (1/500 rabbit GFAP from DAKO) at 4 °C overnight. Fluorescent secondary antibodies (Vector Labs) at a 1:200 dilution were applied for an hour at RT and immunoreactivity detected under fluorescence using DAPI as counterstaining. Coverslips from control and experimental conditions were stained side by side in a single and double labeling paradigm using the same batch of antibodies.

Counts of TUJ-1 and GFAP positive cells were determined by single or double labeling for glial and neuron cells in at least 3 coverslips for the condition from 3 independent experiments.

All histological and immunohistochemical images were acquired from a Zeiss Axioplan 2 D1 microscope equipped with a Tucsen 5.0 ICE digital camera (China).

### Statistical analysis

Results from flow cytometry analysis are reported as median ± 25 percentile of the median from at least 9 independent experiments. We tested whether the median of the variable was affected by the DMEM-GF medium with respect to the DMEM-CM experiments for statistical significance using *U* of Mann Whitney non-parametric analysis. A value of p less than 0.05 was considered significant.

### Ethical approval

All the procedures involving human participants adhered to the ethical standards of the institutional and national research committees and to the 1964 Helsinki Declaration and its later amendments or comparable ethical standards.

## Results

### Culturing cells from explants of human olfactory mucosa

**Figure 1:**
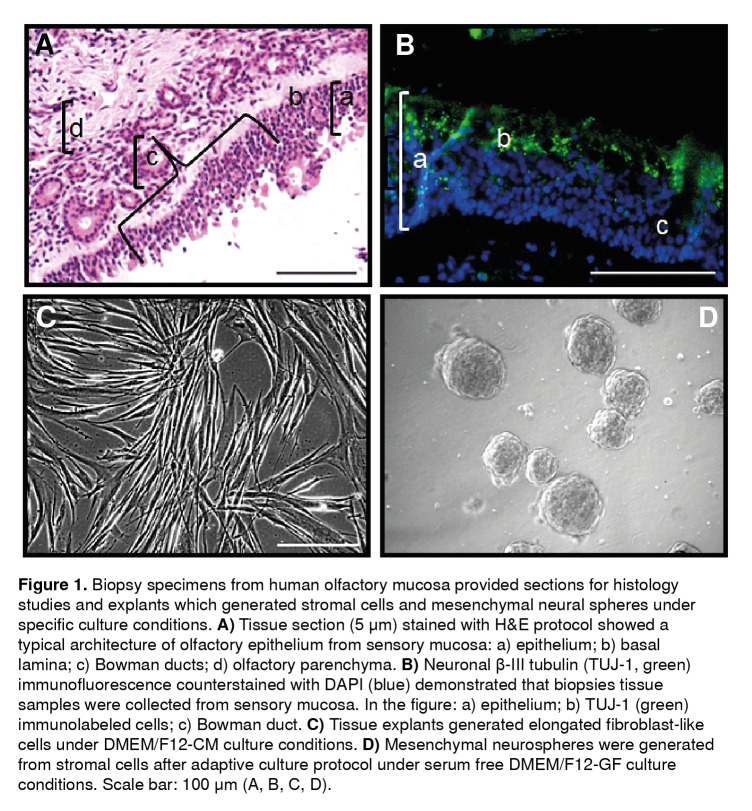
Biopsy specimens from human olfactory mucosa provided sections for histology studies and explants which generated stromal cells and mesenchymal neural spheres under specific culture conditions. **A)** Tissue section (5 µm) stained with H&E protocol showed a typical architecture of olfactory epithelium from sensory mucosa: a) epithelium; b) basal lamina; c) Bowman ducts; d) olfactory parenchyma. **B)** Neuronal (3-III tubulin (TUJ-1, green) immunofluorescence counterstained with DAPI (blue) demonstrated that biopsies tissue samples were collected from sensory mucosa. In the figure: a) epithelium; b) TUJ-1 (green) immunolabeled cells; c) Bowman duct. **C)** Tissue explants generated elongated fibroblast-like cells under DMEM/F12-CM culture conditions. **D)** Mesenchymal neurospheres were generated from stromal cells after adaptive culture protocol under serum free DMEM/F12-GF culture conditions. Scale bar: 100 µm (A, B, C, D).

A typical cytoarchitecture of pseudostratified epithelium lying on a basal lamina was observed from tissue sections under H&E staining protocol (figure 1A). Bowman secretory components of the *lamina propia* occurred underneath the basal lamina (figure 1A). The detection of the expression of B-III tubulin protein using immunofluorescence protocols was suggestive of immature neurons immunolabeled with TUJ-1 antibody (figure 1B). This observation confirmed that the biopsy of the tissue sample was collected from the sensory epithelium of the olfactory nasal cavity.

Explants from human olfactory mucosa biopsies were seeded on cell culture plates with regular medium (DMEM/F12-CM). After 48 h, cells migrated from explants and generated a stroma that reached confluence after 10 to 15 days. A predominant population of fibroblast-like cells was observed migrating from explants (figure 1C). In subsequent experiments, we studied the phenotypic markers and the differentiation potential of these stromal cells under (DMEM/F12-CM) culture conditions, as well as the adaptive response of stromal cells under free-serum medium and we found that stromal cells proliferated as mesenchymal neurospheres (figure 1D). Then, we identified their potential of differentiation to neural cells under the conditions provided by culture medium supplemented with growth factors (DMEM/F12-GF).

### Description of human olfactory mucosa-adherent cells by flow cytometry methodology

**Figure 2:**
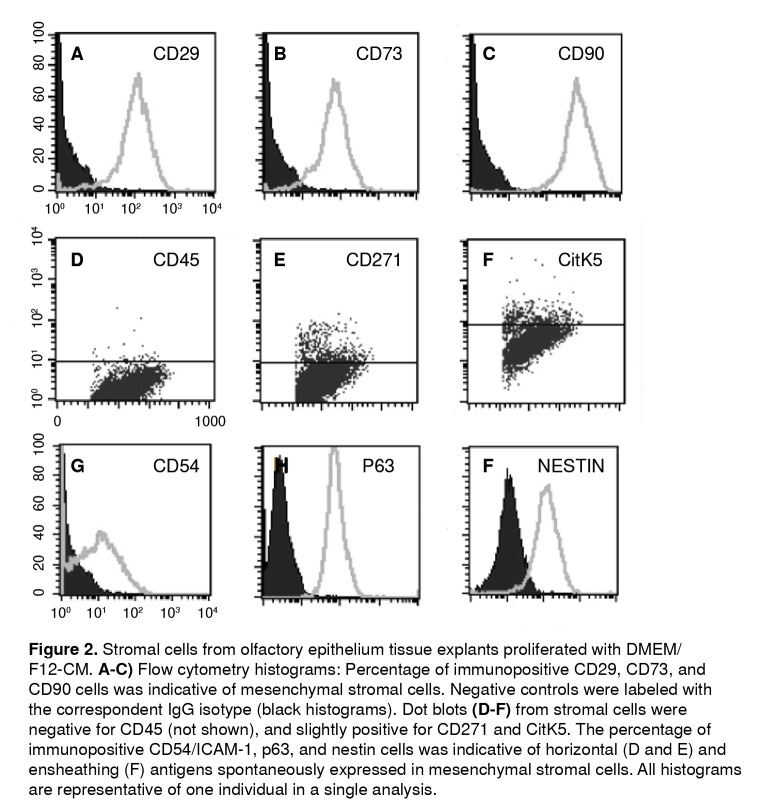
Stromal cells from olfactory epithelium tissue explants proliferated with DMEIW F12-CM. **A-C)** Flow cytometry histograms: Percentage of immunopositive CD29, CD73, and CD90 cells was indicative of mesenchymal stromal cells. Negative controls were labeled with the correspondent IgG isotype (black histograms). Dot blots **(D-F)** from stromal cells were negative for CD45 (not shown), and slightly positive for CD271 and CitK5. The percentage of immunopositive CD54/ICAM-1, p63, and nestin cells was indicative of horizontal (D and E) and ensheathing (F) antigens spontaneously expressed in mesenchymal stromal cells. All histograms are representative of one individual in a single analysis.

Stromal cells from human olfactory mucosa tissue explants were cultured with regular medium (DMEM/F12-CM). We determined the expression of CD29, CD73, and CD90 proteins, well established MSC markers, using the flow cytometry technique (figure 2 A-C). Furthermore, in the same population of cells, we also determined the expression of CD54 (figure 2D) and p63 (figure 2E), which was suggestive of the expression of horizontallike basal cells phenotypic markers. Besides, in this cultured population, we also determined nestin (figure 2F) and GFAP^+^ (not shown) immunopositive cells, which are proteins commonly expressed in ensheathing/neural-like progenitors. Altogether, these results were suggestive of a mesenchymal stromal cell population that co-expressed proteins from olfactory precursors and neural progenitor cells. Interestingly, less than 7% of cells were positive for CD271 (p75NGFr) and cytokeratin 5. It should be noticed that the highest percentage of cells determined by flow cytometry analysis was corresponding with the expression of MSC proteins. As a consequence, findings of horizontal-like basal and ensheathing cell protein markers were suggestive of the concurrent expression of these markers in the MSC population.

**Figure 3:**
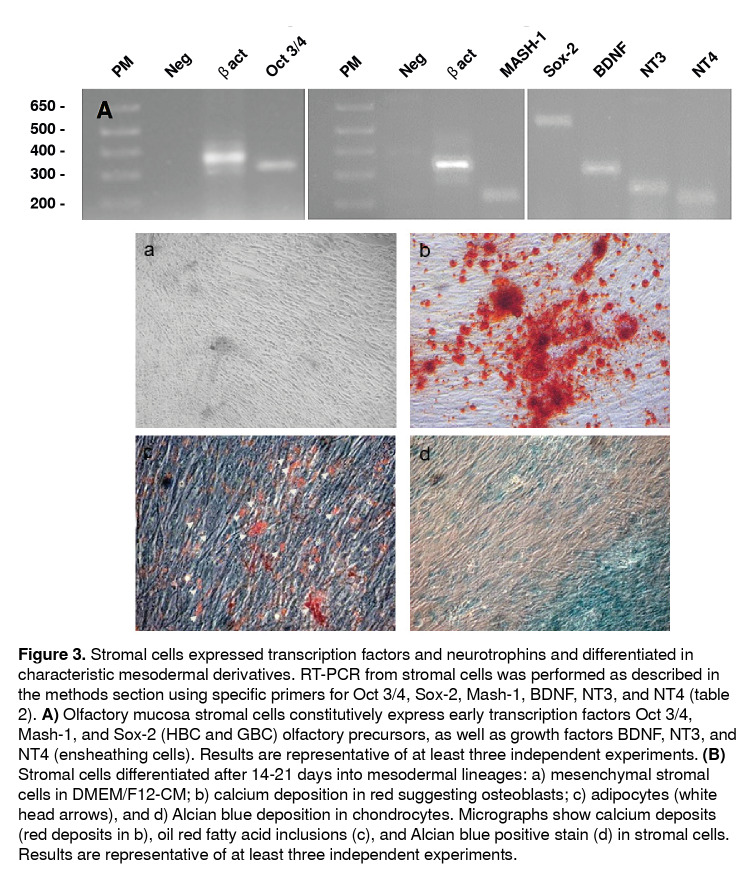
Stromal cells expressed transcription factors and neurotrophins and differentiated in characteristic mesodermal derivatives. RT-PCR from stromal cells was performed as described in the methods section using specific primers for Oct 3/4, Sox-2, Mash-1, BDNF, NT3, and NT4 (table 2). **A)** Olfactory mucosa stromal cells constitutively express early transcription factors Oct 3/4, Mash-1, and Sox-2 (HBC and GBC) olfactory precursors, as well as growth factors BDNF, NT3, and NT4 (ensheathing cells). Results are representative of at least three independent experiments. **(B)** Stromal cells differentiated after 14-21 days into mesodermal lineages: a) mesenchymal stromal cells in DMEM/F12-CM; b) calcium deposition in red suggesting osteoblasts; c) adipocytes (white head arrows), and d) Alcian blue deposition in chondrocytes. Micrographs show calcium deposits (red deposits in b), oil red fatty acid inclusions (c), and Alcian blue positive stain (d) in stromal cells. Results are representative of at least three independent experiments.

In agreement with these findings, we established by PCR methodology the expression of transcription factors (Oct 3/4, *mash-1,* and Sox 2) and neurotrophins (BDNF, NT3, and NT4), commonly associated with sustentacular, HBC, GBC, and ensheathing/neural-like progenitor cells in the adherent population from human olfactory mucosa tissue biopsies (figure 3A). Altogether, these results confirmed a spontaneous co-expression of olfactory and neural progenitor protein markers in the MSC cultured population. In agreement with data from flow cytometry studies, further functional assays demonstrated that adherent MSCs may differentiate under well-established specific culture conditions into osteoblasts, chondroblasts, and adipocytes, respectively (figure 3B-D).

**Figure 4:**
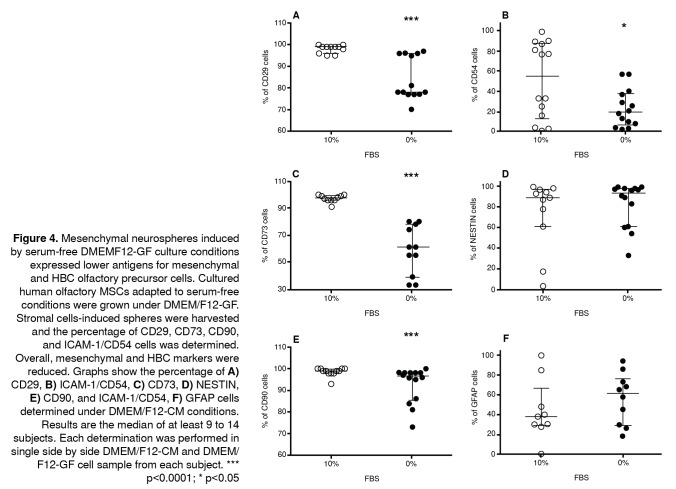
Mesenchymal neurospheres induced by serum-free DMEMF12-GF culture conditions expressed lower antigens for mesenchymal and HBC olfactory precursor cells. Cultured human olfactory MSCs adapted to serum-free conditions were grown under DMEM/F12-GF. Stromal cells-induced spheres were harvested and the percentage of CD29, CD73, CD90, and ICAM-1/CD54 cells was determined. Overall, mesenchymal and HBC markers were reduced. Graphs show the percentage of **A)** CD29, **B)** ICAM-1/CD54, C) CD73, **D)** NESTIN, **E)** CD90, and ICAM-1/CD54, **F)** GFAP cells determined under DMEM/F12-CM conditions. Results are the median of at least 9 to 14 subjects. Each determination was performed in single side by side DMEM/F12-CM and DMEM/ F12-GF cell sample from each subject. "* p<0.0001; * p<0.05

### Growth of stromal cells in spheres induced by serum-free DMEM/F12 GF culture medium

We investigated the capacity of MSCs from human olfactory mucosa to generate mesenchymal neurospheres. For this purpose, confluent monolayers of MSC under DMEM/F12-CM conditions were adapted to serum-free DMEM/ F12 conditions for 5 days. Then, cells were harvested and cultured in serum-free DMEM/F12 medium supplemented with growth factors (DMEM/F12-GF). Under these culture conditions, cells showed noticeable phenotypic changes without decreasing cell viability (figure 1D). After 3 to 4 days, cells grew to form spheres, which were rather similar to neurospheres from neural progenitor cells. The same pattern of cell growth persisted through several passages in DMEM/F12-GF.

### Descriptive phenotypical characterization of mesenchymal neurospheres obtained from human olfactory mucosa MSCs cultured in DMEM/F12-GF

Mesenchymal neurospheres induced by serum-free adapted stromal cells (at least 9 subjects) under DMEM-GF culture conditions were harvested, dissociated, and analyzed using the flow cytometry protocol. Under DMEM/ F12-CM (10% FBS) culture conditions, the median of the percentage of cells expressing mesenchymal stromal proteins (CD29, CD73, and CD90) was around 98% with low variability. In contrast, we observed the reduction of the expression of MSC proteins (p<0.0001) (figure 4 A,C,E) (table 3). Otherwise, the HBC protein marker ICAM-1/CD54 was highly variable, although we found a significant reduction in the median of the percentage of cells expressing the ICAM-1/CD54 phenotypic marker (p<0.05) (figure 4B) (table 3).

**Table 3:** Expression of protein markers for human olfactory mucosa MSCs

DMEM/F12 CM					DMEM/F12-GF	
	n1/n2	Median1 (10% FBS)	Percentile 25%	Median2 (0% FBS)	Percentile 25%	U Mann-Whitney/p
MSC						
CD29	11/13	99	96	78	77	11.5/0.0001
CD73	10/11	97.5	96	61	39	0/0.0001
CD90	12/14	99	98	96.5	85.5	26.5/0.0001
HBC						
CD54	14/14	55	13	19.5	7	59.5/0.039
Ensheathing cells						
Nestin	11/14	89	61	93.5	60.7	65/0.26
GFAP	9/10	38	29	61.5	29	35/0.22

n: Number of subjects

U Mann-Whitney/p: statistical value/probability

Noticeably, the percentage of nestin^+^ and GFAP^+^ cells was not modified under this condition (figure 4 D and F). Overall protein expression of HBC-like and ensheating cell-like proteins was highly variable in the cultured population with DMEM/F12-CM and DMEM/F12-GF. However, these findings were suggestive of the modulation of MSC and HBC-like protein expression metabolically induced by serum-free DMEM-GF culture conditions with the preservation of nestin and GFAP proteins, which suggests the stability of the neural progenitor phenotype.

### Differentiation of cells from mesenchymal neurospheres in human olfactory mucosa MSCs induced by DMEM-GF

**Figure 5:**
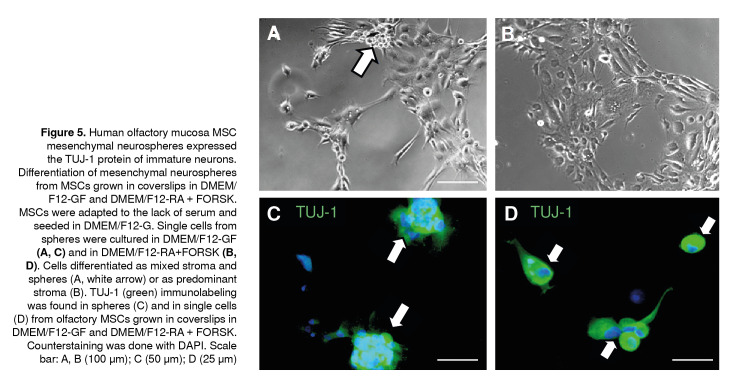
Human olfactory mucosa MSC mesenchymal neurospheres expressed the TUJ-1 protein of immature neurons. Differentiation of mesenchymal neurospheres from MSCs grown in coverslips in DMEM/F12-GF and DMEM/F12-RA + FORSK. MSCs were adapted to the lack of serum and seeded in DMEM/F12-G. Single cells from spheres were cultured in DMEM/F12-GF (A, **C)** and in DMEM/F12-RA+FORSK **(B, D).** Cells differentiated as mixed stroma and spheres (A, white arrow) or as predominant stroma (B). TUJ-1 (green) immunolabeling was found in spheres (C) and in single cells (D) from olfactory MSCs grown in coverslips in DMEM/F12-GF and DMEM/F12-RA + FORSK. Counterstaining was done with DAPI. Scale bar: A, B (100 um); C (50 µm); D (25 µm)

We examined the capacity of cells from spheres induced by using DMEM/ F12-GF to differentiate into early neurons (TUJ-1). For this purpose, single cells from spheres were seeded on PDL-coated coverslips and cultured in DMEM/F12-GF (figure 5A, C) or DMEM/F12 RA+FOSK) for 7 days (figure 5B, D). After seeding, single cells became attached to the substrate in both culture conditions generating spheres attached to the PDL (figure 5A, arrow) or a monolayer with medium to high complexity (figure 5B). We determined a strong expression of TUJ-1 in both culture conditions (figure 5C, D). Interestingly, in both experimental conditions, the expression of TUJ-1 was found both in mesenchymal neurospheres (figure 5C, arrows) and in single cells (figure 5D). We found no differences between the number of TUJ-1 immunopositive cells in any of the experimental conditions (results not shown).

**Figure 6:**
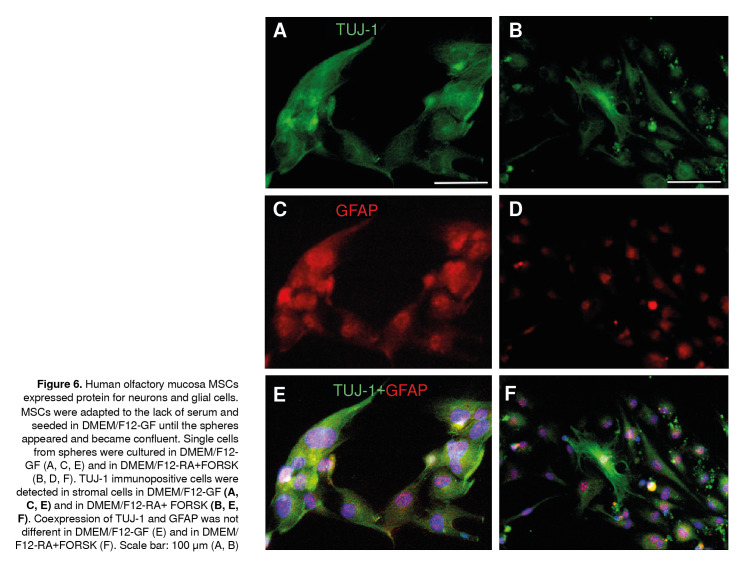
Human olfactory mucosa MSCs expressed protein for neurons and glial cells. MSCs were adapted to the lack of serum and seeded in DMEM/F12-GF until the spheres appeared and became confluent. Single cells from spheres were cultured in DMEM/F12-GF (A, C, E) and in DMEM/F12-RA+FORSK (B, D, F). TUJ-1 immunopositive cells were detected in stromal cells in DMEM/F12-GF (A, **C, E**)and in DMEM/F12-RA+ FORSK **(B, E, F).** Coexpression of TUJ-1 and GFAP was not different in DMEM/F12-GF (E) and in DMEM/ F12-RA+FORSK (F). Scale bar: 100 µm (A, B)

Then, we asked ourselves whether single cells grown under DMEM/F12-GF and DMEM/F12 RA + FORSK conditions would differentiate in neurons or glial cells. To address this question, the expression of TUJ-1 and GFAP proteins was examined after cells were plated and cultured for 7 days in culture mediums as quoted above. We determined TUJ-1 expression in cells under both conditions whereas GFAP expression was co-localized with comparable expression of TUJ 1 under DMEM/F12-GF (figure 6A, C, E); unexpectedly, under DMEM/F12 RA+ FORSK conditions, the expression of GFAP was restricted to the nuclear area (figure 6B, D, F). These findings are suggestive of two reservoirs of GFAP protein in the MSC population. As a consequence, there was no co-localization of both immunomarkers; however, the counts of double-labeled cells were not different under these culture conditions (not shown), which suggests that either DMEM/F12-GF or DMEM/ F12 RA+FORSK may induce differentiation to TUJ-1 immunopositive cells from mesenchymal neurospheres. However, the ectopic expression of GFAP was promoted by DMEM/F12 RA+FORSK.

## Discussion

In this study, we established culture conditions for biopsies of human olfactory mucosa without pretreatment with enzyme proteases and we observed a predominant monolayer of stromal cells under DMEM/F12-CM culture conditions whereas using a serum-free adaptive protocol, mesenchymal neurospheres induced by DMEM/F12-GF were isolated from the cell population. After proliferation and expansion of stromal cells, they were further identified as MSCs by flow cytometry following the ISSCR guidelines ^13^.

As far as we know, this is the first demonstration of MSCs from human olfactory mucosa spontaneously expressing antigens of olfactory basal progenitors and ensheathing/neural-like progenitor cells. Previous results have also shown that mice MSCs from bone marrow also expressed nestin (100%), although BIII-tubulin and neurofilaments expressed more than 15% altogether ^8^. In contrast, we determined more than 80% of nestin-immunopositive cells, around 90% of P63, 50% of ICAM-1/CD54, and 40% of GFAP. These findings suggested that human olfactory mucosa does have a pro-neural-like basal condition in the concurrent population associated with typical phenotypes found in the olfactory mucosa, i.e., HBC, GBC, sustentacular, and ensheathing cells.

Our results suggest that *ex vivo* explants from olfactory mucosa contain stromal cell components expressing phenotypes of the primary tissue, e.g., olfactory precursors, ensheathing/neural-like progenitor and mesenchymal stromal cells of the olfactory niche. In addition, these findings also suggest that the richness of stromal cell phenotypic markers determined under this culture condition is indicative of a complete functional explant preparation of human olfactory epithelium.

Following our tissue collection protocol, samples examined had a characteristic well-structured cytoarchitecture of the olfactory epithelium as shown by H&E staining. Furthermore, the expression of the TUJ-1 antigen in the tissue sample was indicative of immature neurons in the olfactory neuroepithelium. These findings confirm that the tissue samples under study were collected from upper turbinates where constitutive neuronal maturation and turnover actively contribute to the morphology and complexity of the olfactory sample. Additionally, these results are in agreement with the detailed cellular characterization of autopsied human olfactory mucosa samples recently reported ^1^^,^^2^^,^^7^^,^^11^.

Explants cultured under DMEM/F12-CM conditions, proliferating cells migrating from explants, and a stromal monolayer of cells expressing CD29^+^, CD73+, CD90+, CD271+- and negative for the CD45^-^ antigen are established markers of MSCs. We also determined a reduced population of CD271 (p75, NGFr) and cytokeratin 5. Noticeably, the finding of ICAM-1/CD54+ ^5^^,^^35^ and p63^+ (^^6^^,^^7^, as well as nestin^+^ and GFAP^+^ immunopositive cells in the growing population, are indicative of the plethora of proteins expressed in the olfactory stromal cells characteristic of the mucosa, e.g., GBC, HBC, and ensheathing/ neural-like cells.

Based on these results, we can assert that the human olfactory MSCs expressed mRNA messages for Oct 3/4, Sox-2, and *mash-1* transcription factors, which have been associated with olfactory basal cells that participate in the renewal of olfactory epithelium and bulb interneuron specification, migration, and differentiation. Besides, mRNA messages were found for the BDNF, NT3, and NT4 growth factors. For instance, the expression of neurotrophins in the cultured population is suggestive of the survival signaling typical of ensheathing/neural-like progenitor cells in olfactory mucosa tissues.

In previous studies, spontaneous neural transdifferentiation has been suggested for human ^10^ and rodents ^8^^,^^36^ bone marrow MSCs. For instance, MS spontaneously expressed nestin under serum-free conditions. In contrast, an MS profile as the one shown in this study was described for cultured cells isolated after enzymatic digestion and dissection of the *lamina propria*^10^^,^^31^. Although the expression of ICAM-1/CD54+ immunopositive cells was also determined in the cultured samples, which suggests that the HBC antigen may be coexpressed in the BM cultured population, these findings were not discussed ^10^. Furthermore, the CD105 plasma membrane marker, a well-accepted MS marker, seems not to be strongly expressed in olfactory mucosa MSCs, as we also determined ^37^.

In this study, an HBC-like antigen was identified using well-characterized antibodies tested for the *in vivo* expression of CD54+/ICAM-1 and p63, a transcription factor established by previous reports ^5^^-^^7^^,^^20^. The finding of ICAM-1/CD54+ and p63+ transcription-factor immunopositive cells in our experimental paradigm is the first demonstration of *in vitro* proliferation of human mesenchymal stromal cells spontaneously expressing HBC antigens and ensheathing cells. Seminal work by Murrell, *et al.*^31^, demonstrated multipotent stem-like cells in the *lamina propria* and olfactory epithelium after a detailed and extensive analysis to establish the source and origin of stem cells. Their results suggested that neural-like stem cells occurred in both neuroepithelium and *lamina propria,* thus turning them inconclusive. In support of our findings, we determined a high percentage of the mesenchymal stem, olfactory precursors, and ensheathing/neural-like phenotypic markers. The expression of nestin^+^ cells in stroma and spheres under DMEM/F12-CM and -GF conditions, as well as our results, support the neurogenic potential of these cells.

Although the multipotency of mesenchymal stem cells has been clearly established for decades, not all of those isolated from different tissues are equally functional ^12^. For instance, previous reports have shown that olfactory mucosa stromal cells isolated from *lamina propria* reduced their potential to differentiate in mesodermal tissues ^10^. In contrast, in this study, the olfactory mucosa mesenchymal stromal cells under *in vitro* specific culture conditions differentiated in osteoblasts, chondrocytes, and adipocytes, which supports the multipotency already described for olfactory mucosa MSCs.

Given that neural stem cells classically proliferate and self-renew as neurospheres and that it has been shown that the lack of serum stimulates the growth of stromal cells as spheres, we adapted stromal cells to this condition ^38^^,^^39^. We performed an adaptive experiment where cells remained in DMEM/F12-CM and 10% FBS until confluency, and then they were kept in DMEM/F12-0% FBS. Finally, stromal cells were collected and passaged to DMEM/F12-GF to promote proliferation as neurosphere-like spheres. Under this condition, clonal spheres appeared after 3 to 5 DIV.

As a result of the DMEM/F12 GF culture condition, the expression of MS phenotypic markers was reduced whereas the expression of both nestin and GFAP was not affected as regards DMEM/F12-CM. It should be noted that although nestin antigens seems ubiquitous and not specific of neural stem cells, as they are also transiently expressed in other cell progenitors such as muscle, human bone marrow mesenchymal stromal cells, and some epithelial derivatives, they are recognized as associated with neural progenitors in neurogenic niches of the central nervous system. High expression of nestin and GFAP in human olfactory mucosa at low passages, as we have determined, support the presence of constitutive ectodermal neurogenic-like progenitors in the stromal cell population of olfactory mucosa samples.

Our findings are the first evidence of highly expressed nestin^+^ cells under DMEM/F12-CM and in DMEM/F12-GF culture conditions. In contrast, other reports have shown a medium to low expression of nestin, as well as no expression of GFAP positive cells ^32^. In our experimental model, we obtained the proliferation of stromal cells and spheres and the expression of an ensheathing/neural-like progenitor phenotype induced by the DMEM/F12-CM or -GF medium.

The differentiation of MSC in TUJ-1+ immature neurons from human olfactory mucosa mesenchymal neurospheres in DMEM/F12-GF evidence the importance of this experimental approach in therapeutics and its possible applications in regenerative medicine.

Here we have shown that human olfactory mucosa MSCs spontaneously expressed proteins associated with olfactory precursors, such as HBC and GBC, and ensheathing cells. The expression of neural markers has been reported on other sources MSCs ^8^^,^^40^. It may be that cells co-expressing MSC and neural progenitors may represent a special population with a specific pattern for neural differentiation.

In brief, our work demonstrated mesenchymal stromal cells displaying phenotypes of olfactory progenitors and ensheathing cells that downmodulated membrane receptors in the DMEM-F12/GF medium. We have also shown that human olfactory mucosa contains a stromal mesenchymal population responsive to the lack of serum which differentiates to neurons and glial cells. These results support the principle of the differentiation of stromal mesenchymal cells to neurons, which can be used in research and has the potential for regenerative therapies in the clinic.
